# Analysis of positional obstructive sleep apnea features in 12,000 patients at a sleep center

**DOI:** 10.1007/s11325-026-03755-3

**Published:** 2026-07-04

**Authors:** Cíntia Felicio Adriano Rosa, Karina Angélica Soto Chillcce, Michel Burihan Cahali, Marcela Souza Boldt, Victor Henrique Dominiak Soares, Ordival Augusto Rosa, Pedro Henrique Vicari Passos, Matheus Roberto Schetz Alves, Enrico Guido Oliveira Minniti, Jefferson Ernani Rodrigues, Giuliana Rita Wolski Ribas

**Affiliations:** 1 Fellowship Program, Hospital IPO, Av. República Argentina, 2069, Água Verde, Curitiba, PR CEP 80620-010 Brazil; 2 Department of Otolaryngology, Faculdade de Medicina, Universidade de Sao Paulo, Sao Paulo, Brazil; 3 Fellowship Program in Otorhinolaryngology, Paranás Institute of Otorhinolaryngology, Curitiba, Brazil; 4 Department of Otolaryngology, Universidade de São Paulo (associated with Hospital das Clínicas HCFMUSP and Faculdade de Medicina), São Paulo, Brazil; 5 Federal University of Paraná, Curitiba, Brazil; 6 Otorhinolaryngology Department / Fellowship Program, Hospital IPO (Hospital Paranaense de Otorrinolaringologia), Curitiba, Brazil; 7 Pontíficia Universidade Católica do Paraná, Curitiba, Brazil; 8 Physical Education, Curitiba, Brazil; 9 Biomedical scientist and master’s student in Clinical Research, Federal University of Paraná (UFPR), Curitiba, Brazil

**Keywords:** Positional obstructive sleep apnea, Obstructive sleep apnea, Polysomnography, Phenotype

## Abstract

**Purpose:**

This study aimed to determine the prevalence of Positional Obstructive Sleep Apnea (POSA) and evaluate clinical and polysomnographic characteristics.

**Methods:**

Retrospective analysis of patients undergoing polysomnography (September 2017–August 2023), classified as POSA or non-POSA and as supine-predominant (p-POSA) or supine-exclusive (e-POSA).

**Results:**

Among 12,036 patients with OSA, 66.3% had POSA and 27.8% had e-POSA. Compared with non-POSA, POSA patients had lower AHI (24.1 vs. 44.4 events/h, *p* < 0.001), lower BMI (29.2 vs. 31.4 kg/m², *p* < 0.001), and were slightly younger. No sex differences were observed for POSA, although e-POSA was more frequent in women (*p* < 0.001). After adjustment for AHI, POSA remained characterized by shorter sleep and REM latency, longer total sleep time, higher sleep efficiency, greater proportion of N3, improved oxygenation (higher mean SpO₂ and lower T90), and lower arousal index (all *p* < 0.01). NREM oxygen saturation and total arousals were not significantly different. Significant group × AHI interactions (*p* < 0.05) indicated that these differences were more pronounced at lower AHI levels and attenuated with increasing severity. Among POSA patients, 32.0% had REM OSA (REM-AHI ≥ 2× NREM-AHI), while nearly 70% of REM OSA exhibited POSA.

**Conclusion:**

POSA is highly prevalent and characterized by lower BMI and a more favorable sleep and oxygenation profile independent of AHI. Differences in POSA, although small, were more evident in milder disease and diminished with increasing severity. The overlap with REM OSA was asymmetric, with positional dependence more frequent in REM OSA than the converse. These findings highlight the heterogeneity of POSA and need for individualized management.

## Introduction

Obstructive sleep apnea (OSA) is a disorder characterized by the repeated partial or complete closure of the upper airway during sleep, resulting in intermittent hypoxemia and arousal from sleep [1]. Its consequences include excessive daytime sleepiness, reduced quality of life, cardiovascular diseases, and neurocognitive deficits [2]. It is estimated that approximately one billion adults worldwide, aged 30 to 69 years, may have OSA [3]. Brazil was the third country with the highest number of affected individuals [3].

OSA can be classified as positional (POSA) or non-positional (non-POSA) depending on the occurrence of respiratory events related to body position during sleep. Patients with positional obstructive sleep apnea (POSA) exhibit increased upper airway collapsibility when lying in the supine position compared to the lateral position [4]. Cartwright defined that patients are diagnosed as POSA if apnea-hypopnea index (AHI) is ≥ 5 events/h and at least twice as high while sleeping in the supine position compared to the non-supine position [5]. Subsequently, within this classification, patients can be further subdivided into two groups: supine-exclusive (e-POSA, with non-supine AHI ≤ 5 events/h) or supine-predominant (p-POSA, with non-supine AHI > 5 events/h) [6, 7]. Approximately 50–60% of patients in sleep clinic populations exhibit POSA [4]. According to international literature, patients with POSA tend to be younger, have a lower frequency of snoring, a lower body mass index, and present a milder form of OSA compared to patients with non-positional OSA [8, 9]. The presence of POSA can impact the agreement between tests conducted in sleep laboratories or at home (Home sleep apnea test, HSAT). Patients with greater variations in AHI between in-lab and HSAT also have greater variations in supine AHI [10].

The importance of identifying POSA is directly reflected in treatment. In general, positive airway pressure (PAP) is the primary treatment for moderate to severe OSA. However, its efficacy is limited by poor patient adherence, which is a widely recognized challenge in disease management. Distinguishing between POSA and non-POSA can significantly influence the choice of treatment, as patients with POSA tend to benefit from positional therapy. This therapy involves methods to maintain the patient in non-supine position during sleep [4]. According to the American Academy of Sleep Medicine, positional therapy is an effective secondary therapy or can complement primary therapies for OSA in patients with a lower AHI in the non-supine position compared to the supine position [11].

Therefore, due to this spectrum within OSA classification, the differences in managing patients with POSA, and the lack of data in the literature on the Brazilian population, the aim of this study was to evaluate, in a large clinical sample, the factors associated with POSA and determine its prevalence.

## Methods

From a database of 19,109 polysomnography (PSG) reports conducted at Paraná’s Institute of Otorhinolaryngology (Hospital IPO), exams performed between September 2017 and August 2023 were selected for analysis. Exclusion criteria included age < 18 years, total AHI < 5 events/hour, total sleep time (TST) < 240 min, and missing AHI data for either the supine or non-supine positions. Because precise sleep duration in each body position was unavailable, cases were additionally excluded when total AHI was equal or differed by less than 1 event/hour from the AHI in one body position, and the AHI in the alternate position was 0 events/hour. This pattern indicates that the alternate position contributed negligibly to the overall AHI, likely due to minimal time spent in that position, precluding meaningful positional comparison. An exploratory sensitivity analysis was performed using indirect estimates of supine and non-supine sleep time, derived from total, supine, and non-supine AHI in combination with total sleep time. Cases with invalid or low-reliability estimates (including negative values, zero estimated positional time, or estimated positional time < 30 min) were excluded. Because multiple criteria could apply to the same individual, counts for each exclusion reason are not mutually exclusive. This analysis was conducted to assess the robustness of the findings and was not used as a primary analytical approach. All patients underwent baseline PSG using the Alice digital computerized system (*Philips*^®^). The study was approved by the Research Ethics Committee of our institution (Hospital IPO, Curitiba, Brazil), with CAAE identification on Plataforma Brazil No. 69580723.0.0000.5529.

The following variables were monitored: electroencephalogram with electrodes positioned according to the International 10–20 System, right and left electrooculogram, submental and bilateral tibial electromyogram, electrocardiogram, airflow (nasal cannula and thermistor), respiratory effort (thoracoabdominal bands), arterial oxygen saturation (SaO2), body position, and snoring. Evaluation criteria followed the current American Academy of Sleep Medicine Manual for the Scoring of Sleep and Associated Events [12]. Each exam was analyzed by one of six specialist physicians, all board certified in sleep medicine.

The AHI was calculated considering central, obstructive, or mixed apneas and hypopneas, depending on the effort recorded by thoracoabdominal bands. Apnea was defined as a ≥ 90% reduction in airflow amplitude on the thermistor for ≥ 10 s. Hypopnea was defined as a ≥ 30% reduction in airflow amplitude on the nasal cannula for ≥ 10 s, associated with oxygen desaturation of at least 3% and/or arousal from sleep. The severity of OSA was classified as mild (5 ≤ AHI < 15), moderate (15 ≤ AHI < 30), or severe (AHI ≥ 30). The patients (*n* = 4,343) who underwent PSG during the COVID-19 pandemic did not use the thermistor, in accordance with the recommendations of the local health surveillance agency, and instead, the cannula sensor signal was used for apnea analysis.

In this study, POSA refers specifically to supine-related OSA. Patients were classified into non-POSA or POSA according to Cartwright’s criterion [5]. Patients with POSA were further classified into e-POSA and p-POSA supine-exclusive (e-POSA, with non-supine AHI ≤ 5 events/h) or supine-predominant (p-POSA, with non-supine AHI > 5 events/h) [6, 7]. Associations between patient groups were analyzed considering the following variables: sex, AHI severity categories, age, body mass index (BMI, kg/m^2^), POSA, e- POSA, p-POSA, AHI, AHI-REM (Rapid Eye Movement), AHI-NREM (Non Rapid Eye Movement), supine AHI, non-supine AHI, sleep latency, TST, sleep efficiency, percentage of stages N1, N2, N3, and REM sleep, number of arousals, arousal index, number of obstructive, central, and mixed apneas, percentage of TST spent with oxygen saturation below 90% (T90), average saturation during sleep, and average saturation during REM and NREM sleep.

To explore the overlap between positional and REM OSA, we performed an additional analysis using REM and NREM AHI values. REM OSA was defined using two approaches: a broad definition (REM OSA: REM AHI ≥ 2× NREM AHI) and a stricter definition (REM-Predominant OSA: REM AHI ≥ 2× NREM AHI and NREM AHI < 15 events/hour). This analysis was considered exploratory and was not part of the primary study objectives.

Data was organized in an Excel^®^ spreadsheet and analyzed using IBM SPSS Statistics v.30.0.0. Quantitative variables were described using mean and standard deviation. Quantitative variables were described using mean and standard deviation. Categorical variables were described as absolute and percentage frequencies. Given the large sample size and the approximately symmetric distributions of the quantitative variables, the use of parametric tests was considered appropriate. To compare the groups defined as POSA (yes or no) regarding quantitative variables, the Student’s t-test for independent samples was used. Categorical variables were analyzed using the chi-square test. Effect sizes (e.g., Cohen’s d for t-tests and Cramér’s V for chi-square tests) were estimated for all comparisons. For multiple comparisons, p-values were adjusted using the Bonferroni correction. Given the large sample size (*n* = 12,036), the study had > 90% power to detect small effect sizes (Cohen’s d = 0.1) for group comparisons at α = 0.05. To further explore whether differences between patients with POSA and non-POSA were independent of overall OSA severity, we performed supplementary analyses using linear regression models. Group (POSA vs. non-POSA) was included as the main predictor, and total AHI was entered as a covariate. A group-by-AHI interaction term was included to assess whether the association between OSA severity and each sleep variable differed between groups. These analyses were restricted to polysomnographic variables reflecting sleep architecture, sleep continuity, fragmentation, and oxygenation. Respiratory indices were not included, as they are closely related to both the definition of POSA and total AHI, making such adjustment difficult to interpret. The significance level was set at 0.05 for all analyses.

## Results

**Fig. 1:**
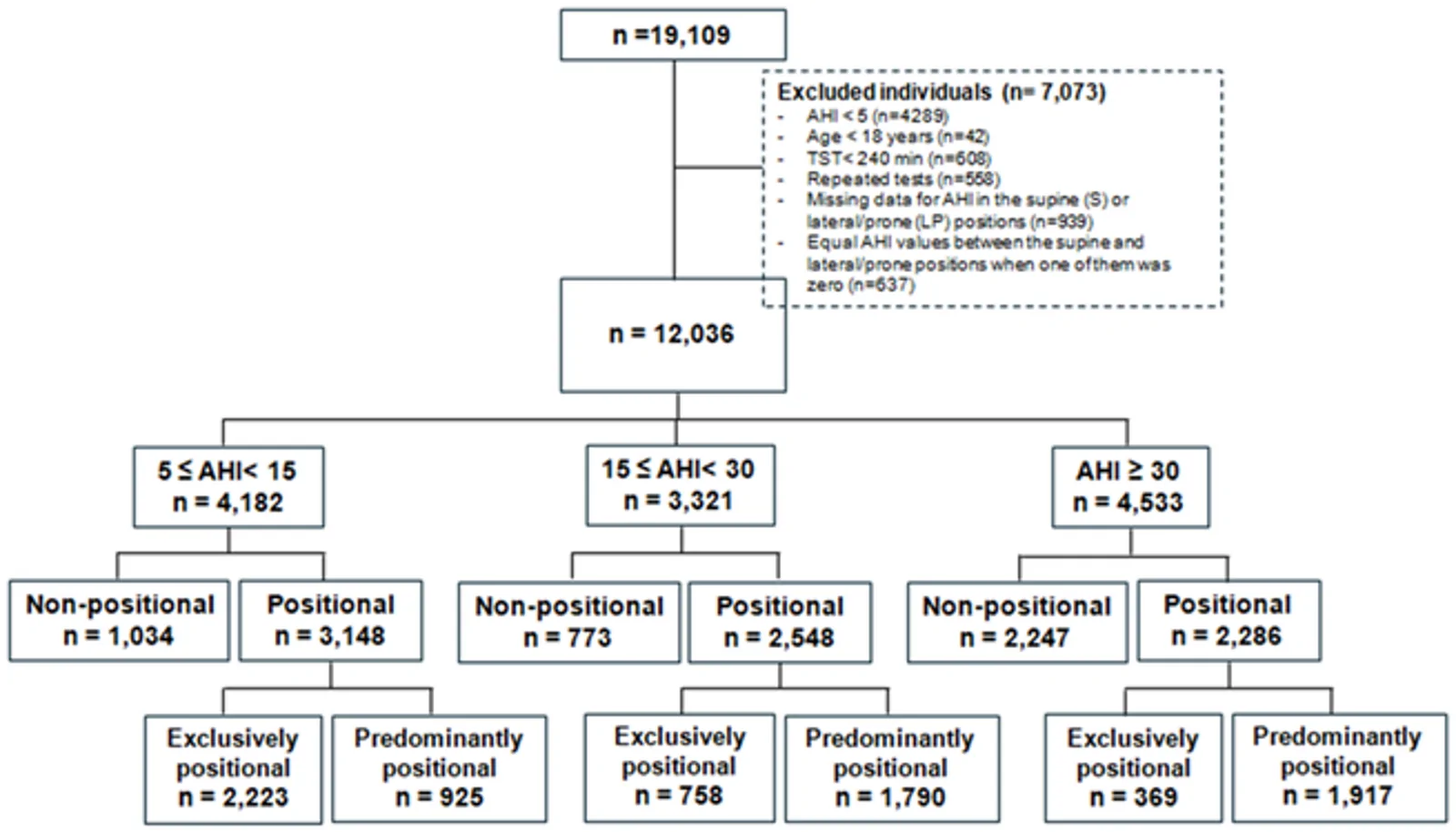
Flowchart of sample selection and categorization by position and OSA severity. Abbreviations: OSA, obstructive sleep apnea; AHI, apnea–hypopnea index; TST, total sleep time; S, supine position; LP, lateral or prone position

Out of 19,109 exams selected from the database, the final sample consisted of 12,036 patients after excluding 7,073 patients based on the criteria outlined in Fig. 1. In a sensitivity analysis, 10,921 patients remained after exclusion of implausible or unreliable estimates. A total of 1,115 patients were excluded from the sensitivity analysis. The main reasons, which were not mutually exclusive, included positional sleep time < 30 min (*n* = 1,113), zero values (*n* = 12), implausible values (*n* = 206), and non-computable values due to mathematical constraints (*n* = 6). The prevalence of supine OSA was 66.7% in the restricted cohort versus 66.3% in the full cohort, and the prevalence of exclusive supine OSA was 29.2% versus 27.8%, respectively.

There was a predominance of male patients (61.6%) compared to female patients (38.4%). The mean age was 47.9 ± 13.5 years, and the mean BMI was 29.9 ± 4.9 kg/m^2^, characterizing a population with overweight or obesity. Regarding OSA severity, 34.7% of patients were classified as mild, 27.6% as moderate, and 37.7% as severe OSA (Table 1). The prevalence of POSA was 66.3% of the sample, with a very similar sex distribution (3,064 females and 4,918 males with OSA presented as POSA, 66.3 versus 66.4%, respectively, *p* = 0.921, Chi-square test). Compared to non-POSA, patients with POSA had significantly lower age (47.6 ± 13.1 vs. 48.3 ± 14.3 years, *p* = 0.012, although the effect size was trivial - Cohen’s *d* = 0.05) and BMI (29.2 ± 4.4 vs. 31.4 ± 5.6 kg/m^2^, *p* < 0.001, with a small-to-moderate effect size - Cohen’s *d* = 0.46, Table 1). In the overall cohort, the prevalence of e-POSA was 27.8%. Positional AHI values of 0 events/hour were observed in 598 patients (5.0%) in the non-supine position and in 8 patients (0.1%) in the supine position.

**Table 1:** Demographic characteristics of patients

Variable	Whole group 12,036 (100%)	POSA 7,982 (66.3%)	Non-POSA 4,054 (33.7%)	*p*-value *	Effect size (Cohen’s d)
Age, years	47.9 (13.5)	47.6 (13.1)	48.3 (14.3)	0.012 †	0,052 (very small)
Male/Female (%)	61.6 / 38.4	61.6 / 38.4	61.5 / 38.5	0.921	-
BMI, mean (SD), kg/m^2^	29.9 (4.9)	29.2 (4.4)	31.4 (5.6)	< 0.001 †	0,455 (small)
Mild OSA (n, %)	4,182 (34.7)	3,148 (39.4)	1,034 (25.5)		
Moderate OSA (n, %)	3,321 (27.6)	2,548 (31.9)	773 (19.1)	< 0.001†**	
Severe OSA (n, %)	4,533 (37.7)	2,286 (28.6)	2,247 (55.4)		

Abbreviations: *POSA* positional obstructive sleep apnea; non-POSA, non-positional obstructive sleep apnea, *BMI* body mass index, *OSA* obstructive sleep apnea, *SD* standard deviation

*Student’s t-test for independent samples (quantitative variables), Fisher’s exact test or Chi-square test (categorical variables) (Bonferroni-adjusted p-values for multiple comparisons), Effect sizes were calculated as Cohen’s *d*

** Mild vs. moderate (*p* < 0.001), mild vs. severe (*p* < 0.001), moderate vs. severe (*p* < 0.001)

† *p* < 0.05

Regarding the polysomnographic findings, compared to non-POSA, patients with POSA showed statistically significant differences with several parameters indicating less severe OSA, including lower mean AHI in both REM and NREM sleep, less time with oxygen saturation below 90%, higher mean arterial oxygen saturation during the night, less sleep fragmentation, greater amount of N3 and REM sleep stages, and higher sleep efficiency (*p* < 0.001 for all, Table 2). As expected, based on the definition of POSA, non-supine AHI differed substantially between groups (10.2 ± 10.7 vs. 41.8 ± 32.2, *p* < 0.001, Table 2) and was not considered an independent finding, whereas supine AHI values were similar. Effect size analysis (Table 3) demonstrated that the magnitude of differences between POSA and non-POSA groups varied widely across polysomnographic parameters. Large effects were found for total AHI (*d* = 0.85). Moderate effects emerged for total and obstructive apnea and hypopnea indices, arousal index, T90, AHI-REM, AHI-NREM and mean nocturnal oxygen saturation. Conversely, small or very small effects were detected for N1–N3 and REM sleep percentages, mixed apneas, mean SpO₂ during NREM, sleep latency, TST, sleep efficiency, and REM latency, suggesting minimal clinical differences. After adjustment for total AHI, several differences between patients with POSA and non-POSA remained statistically significant, particularly in measures of sleep architecture, sleep continuity, and oxygenation parameters. In contrast, some variables, such as NREM oxygen saturation and total arousals, were no longer significantly different between groups after adjustment (Table 4). Overall, POSA was characterized with a more favorable sleep profile, including deeper sleep stages, better oxygenation, and reduced sleep fragmentation. Significant group × AHI interactions were observed for sleep stage distribution (N1, N2, N3, REM), oxygenation parameters (mean SpO₂, REM SpO₂, and T90), and arousal index (p for interaction < 0.05), with differences between POSA and non-POSA being greater at lower AHI levels and progressively attenuated with increasing disease severity.

**Table 2:** Polysomnographic characteristics of the patients, expressed as mean and standard deviation

Variable	Whole group *n* = 12,036 (100%)	POSA *n* = 7982 (66.3%)	Non-POSA 4054 (33.7%)	*p*-value *
AHI, events/h	30.9 (25.8)	24.1 (17.8)	44.4 (32.8)	< 0.001 †
AHI-REM, events/h	35.6 (26.8)	28.9 (22.3)	48.6 (29.8)	< 0.001 †
AHI-NREM, events/h	29.7 (27.1)	22.9 (18.9)	43.1 (34.7)	< 0.001 †
Supine AHI, events/h	48.7 (33.5)	48.4 (31.3)	49.1 (37.3)	0.275
Non-supine AHI, events/h	20.9 (25.5)	10.2 (10.7)	41.8 (32.2)	< 0.001 †
Sleep lat, min	14.6 (18.1)	14.4 (17.2)	15 (19.8)	0.079
REM lat, min	130.5 (76.8)	126.6 (73.8)	138.3 (81.8)	< 0.001 †
TST, min	379.5 (52.6)	381 (52.0)	376.4 (53.6)	< 0.001 †
SE, %	84.4 (9.5)	84.8 (9.3)	83.6 (9.8)	< 0.001 †
N1%	4.6 (4.3)	4.3 (3.6)	5.2 (5.4)	< 0.001 †
N2%	56.0 (11.0)	55.1 (10.3)	57.8 (12.1)	< 0.001 †
N3%	21.2 (8.6)	21.9 (7.9)	19.7 (9.5)	< 0.001 †
REM %	18.2 (6.6)	18.7 (6.4)	17.3 (6.9)	< 0.001 †
Total arousals, n	128.4 (100)	109.3 (69.5)	166 (134.4)	< 0.001 †
Arousal index, events/h	20.8 (15.7)	17.6 (10.8)	27 (20.9)	< 0.001 †
Apnea index, events/h	11.9 (18.9)	7.7 (11.1)	20.2 (26.8)	< 0.001 †
Hypopnea index, events/h	19.1 (14.6)	16.5 (11.5)	24.2 (18.2)	< 0.001 †
Total hypopneas, n	120.2 (93.3)	104.5 (74.7)	151 (115.9)	< 0.001 †
Total obstructive apneas, n	54.7 (97.3)	35 (57.6)	93.4 (139)	< 0.001 †
Total central apneas, n	9.0 (23.5)	7.7 (19.4)	11.5 (29.7)	< 0.001 †
Total mixed apneas, n	11.4 (37.2)	6.1 (19.7)	21.8 (56.3)	< 0.001 †
T90, %	8.6 (16.1)	5.5 (11.8)	14.7 (21.0)	< 0.001 †
WASO, min	56.1 (39.0)	54.4 (38.1)	59.2 (40.4)	< 0.001 †
O_2_, %	92.9 (3.5)	93.6 (2.3)	91.7 (4.8)	< 0.001 †
O_2_ REM, %	92.8 (3.6)	93.5 (2.2)	91.5 (5.1)	< 0.001 †
O_2_ NREM, %	86.5 (10.2)	87.7 (8.2)	84.2 (12.8)	< 0.001 †

Abbreviations: *Lat*, latency, *Min* minute, *POSA* positional obstructive sleep apnea, *AHI* apnea-hypopnea index, AHI-REM / AHI-NREM, AHI during rapid eye movement (REM) or non-REM (NREM) sleep, *TST* total sleep time, *SE* sleep efficiency, N1/N2/N3/REM, percentage of stage N1/N2/N3/REM sleep; T90, total sleep time (%) with oxygen saturation below 90%, *WASO* wake after sleep onset, O_2_ / O_2_ REM / O_2_ NREM, average oxygen saturation during sleep / REM sleep / NREM sleep

*Student’s t-test for independent samples, *p* < 0.05 † *p* < 0.05

**Table 3:** Effect size (Cohen’s *d*) for differences between POSA and non-POSA groups

Variable	Cohen’s d	95%CI	Effect size interpretation
AHI, events/h	0.846	0.807; 0.886	Large
AHI-REM, events/h	0.785	0.746; 0.824	Moderate
AHI-NREM, events/h	0.795	0.756; 0.834	Moderate
Supine AHI, events/h	0.022	-0.016; 0.060	Very Small
Non-supine AHI, events/h	1.532	1.489; 1.574	Very Large
Sleep lat, min	0.035	-0.002; 0.073	Very Small
REM lat, min	0.153	0.115; 0.191	Very Small
TST, min	-0.088	-0.125; -0.050	Very Small
SE, %	-0.122	-0.160; -0.084	Very Small
N1%	0.202	0.164; 0.240	Small
N2%	0.253	0.215; 0.291	Small
N3%	-0.259	-0.297; -0.221	Small
REM %	-0.216	-0.254; -0.178	Small
Total arousals, n	0.588	0.549; 0.626	Moderate
Arousal index, events/h	0.627	0.588; 0.666	Moderate
Apnea index, events/h	0.697	0.658; 0.736	Moderate
Hypopnea index, events/h	0.546	0.508; 0.585	Moderate
Total hypopneas, n	0.512	0.474; 0.551	Moderate
Total obstructive apneas, n	0.626	0.587; 0.664	Moderate
Total central apneas, n	0.163	0.125; 0.201	Very Small
Total mixed apneas, n	0.431	0.392; 0.469	Small
T90, %	0.590	0.552; 0.629	Moderate
WASO, min	0.123	0.085; 0.161	Very Small
O_2_, %	-0.558	-0.596; -0.519	Moderate
O_2_ REM, %	-0.574	-0.613; -0.536	Moderate
O_2_ NREM, %	-0.347	-0.385; -0.309	Small

Abbreviations: *Lat* latency, *Min* minute, *POSA* positional obstructive sleep apnea, *AHI* apnea-hypopnea index, AHI-REM / AHI-NREM, AHI during rapid eye movement (REM) or non-REM (NREM) sleep, *TST* total sleep time, *SE* sleep efficiency, N1/N2/N3/REM, percentage of stage N1/N2/N3/REM sleep, T90, total sleep time (%) with oxygen saturation below 90%; WASO, wake after sleep onset; O_2_ / O_2_ REM / O_2_ NREM, average oxygen saturation during sleep / REM sleep / NREM sleep

95%CI: 95% confidence interval *Confidence intervals that include zero

Effect size (Cohen’s *d*) expresses the magnitude of mean differences between POSA and non-POSA groups in standard deviation units. Values were computed as (mean non-POSA − mean POSA) / SD pooled, where positive *d* indicates higher values in the non-POSA group and negative *d* indicates higher values in the POSA group. Effect size magnitude was interpreted according to Sawilowsky:| |*d*| <0.20 very small, |*d*| = 0.20–0.49 small, 0.50–0.79 moderate, 0.80–1.19 large, and ≥ 1.20 very large

**Table 4:** Adjusted associations between positional phenotype and sleep and oxygenation parameters, including group-by-AHI interaction

Variable	POSA (*n* = 7982)	Non-POSA (*n* = 4054)	Adjusted β for POSA*	95% CI	*p*-value	*p* for group×AHI	
Sleep latency (min)	14.4 (17.2)	15.0 (19.8)	-1.11	-1.84 to -0.37	0.003	0.373	
REM latency (min)	126.6 (73.8)	138.3 (81.8)	-4.79	-7.89 to -1.69	0.002	0.389	
Total sleep time (min)	381.0 (52.0)	376.4 (53.6)	4.15	2.01 to 6.29	< 0.001	0.186	
Sleep efficiency (%)	84.8 (9.3)	83.6 (9.8)	0.77	0.39 to 1.16	< 0.001	0.322	
N1 (%)	4.3 (3.6)	5.2 (5.4)	-0.18	-0.35 to -0.01	0.041	0.006	
N2 (%)	55.1 (10.3)	57.8 (12.1)	-0.72	-1.16 to -0.29	0.001	< 0.001	
N3 (%)	21.9 (7.9)	19.7 (9.5)	0.52	0.19 to 0.86	0.002	< 0.001	
REM (%)	18.7 (6.4)	17.3 (6.9)	0.38	0.11 to 0.64	0.005	0.008	
WASO (min)	54.4 (38.1)	59.2 (40.4)	-2.27	-3.84 to -0.69	0.005	0.102	
Mean O₂ saturation (%)	93.6 (2.3)	91.7 (4.8)	0.49	0.37 to 0.61	< 0.001	< 0.001	
O₂ saturation in REM (%)	93.5 (2.2)	91.5 (5.1)	0.60	0.48 to 0.72	< 0.001	< 0.001	
O₂ saturation in NREM (%)	87.7 (8.2)	84.2 (12.8)	0.24	-0.14 to 0.62	0.217	0.487	
T90 (%)	5.5 (11.8)	14.7 (21.0)	-2.21	-2.74 to -1.67	< 0.001	< 0.001	
Total arousals (n)	109.3 (69.5)	166.0 (134.4)	-2.60	-5.53 to 0.34	0.083	< 0.001	
Arousal index (events/h)	17.6 (10.8)	27.0 (20.9)	-0.75	-1.20 to -0.30	< 0.001	< 0.001	

Abbreviations: *Lat* latency, *Min* minute, *POSA* positional obstructive sleep apnea, *AHI* apnea-hypopnea index; N1/N2/N3/REM, percentage of stage N1/N2/N3/REM sleep; T90, total sleep time (%) with oxygen saturation below 90%; WASO, wake after sleep onset; O2 / O2 REM / O2 NREM, average oxygen saturation during sleep / REM sleep / NREM sleep

*Adjusted β for POSA represents the mean difference between the POSA and non-POSA groups after adjustment for total AHI. Linear regression models included group as the main predictor and total AHI as an adjustment variable. An additional model including a group-by-AHI interaction term was fitted for each variable

In post-hoc analyses across AHI categories, patients with AHI ≥ 30 exhibited a lower prevalence of POSA compared with those in the 5–15 and 15–30 ranges (both *p* < 0.001). No significant difference was found between the 5–15 and 15–30 groups (*p* = 0.145, Table 5).

**Table 5:** Proportion of POSA According to AHI Classification (mild, moderate, severe)

POSA	AHI						p*
	5 ≤ AHI < 15		15 ≤ AHI < 30		AHI ≥ 30		
No	1034	24,7%	773	23,3%	2247	49,6%	
Yes	3148	75,3%	2548	76,7%	2286	50,4%	< 0,001
Total	4182	100%	3321	100%	4533	100%	

Abbreviations: *POSA* positional obstructive sleep apnea, *AHI* apnea-hypopnea index

* Chi-square test, *p* < 0,05 (Bonferroni-adjusted p-values for multiple comparisons)

* 5–15 vs. 15–30 (*p* < 0.001), 5–15 vs. ≥ 30 (*p* = 0.145), 15–30 vs. ≥ 30 (*p* < 0.001)

Cramer’s V effect size: 0,262 (small)

Among patients with POSA, 58% exhibited p-POSA and 42% e-POSA. In the female POSA group, e-POSA and p-POSA were similarly present (47.8 versus 52.2%), whereas most (61.7%) in the male POSA group presented as p-POSA, and this difference between sex was significant (*p* < 0.001). Compared to p-POSA, patients with e-POSA were younger (*p* < 0.001) and had a lower BMI (*p* < 0.001). Most patients with mild POSA had e-POSA (70.6%), whereas in moderate and severe POSA most patients had p-POSA (70.3% and 83.9%, respectively, *p* < 0.001 for all, Table 6).

**Table 6:** Types of POSA according to sex, age, body mass index and OSA severity

Variable	e-POSA (*n* = 3,350)	*p*-POSA (*n* = 4,632)	*p*-value
Female (*n* = 3,064), %	47.8	52.2	< 0.001 †^a^
Male (*n* = 4,918), %	38.3	61.7	
Age (mean ± SD), years	46.2 ± 12.9	48.6 ± 13.2	< 0.001 †^b^
BMI, (mean ± SD), kg/m^2^	28.3 ± 4.2	29.8 ± 4.4	< 0.001 †^b^
Mild POSA (*n* = 3,148), %	70.6	29.4	
Moderate POSA (*n* = 2,548), %	29.7	70.3	< 0.001 †^c **^
Severe POSA (*n* = 2,286), %	16.1	83.9	

Abbreviations: e-POSA, exclusive positional obstructive sleep apnea (supine AHI ≥ 2× non-supine AHI and non-supine AHI ≤ 5 events/h); p-POSA, predominant positional obstructive sleep apnea (supine AHI ≥ 2× non-supine AHI with non-supine AHI > 5 events/h), *SD* standard deviation, *BMI* body mass index

^a^ Fisher’ exact test (for sex comparison)

^b^ Student’s t-test for independent samples

^c^ Chi-square test (for xx comparison) (Bonferroni-adjusted p-values for multiple comparisons)

** Mild vs. moderate (*p* < 0.001), mild vs. severe (*p* < 0.001), moderate vs. severe (*p* = 0,145; Holm-Bonferroni)

† *p* < 0.05

There was a substantial but incomplete overlap between positional and REM OSA phenotypes (Table 7). Of the original sample, 11,073 patients had REM sleep time ≥ 30 min and were included in the REM OSA analysis. Among them, 3,427 (30.9%) met the broad REM OSA definition (REM AHI ≥ 2× NREM AHI), and 2,569 (23.2%) met the stricter definition (REM-predominant OSA: REM AHI ≥ 2× NREM AHI and NREM AHI < 15 events/h). In patients with POSA, 32.0% met the broad REM OSA definition, whereas 69.8% of patients with broad definition of REM OSA were classified as POSA. Similar patterns were observed using the stricter definition (REM-predominant OSA).

**Table 7:** Bidirectional overlap between POSA and REM OSA phenotypes

A. POSA and REM OSA		
Phenotype (denominator)	REM OSA (broad)	REM-predominant OSA (strict)
POSA (*n* = 7,465)	2,391 (32.0%)	1,838 (24.6%)
e-POSA (*n* = 3,142)	1,259 (40.1%)	1,151 (36.6%)
B. REM OSA and POSA		
Phenotype (denominator)	POSA	e-POSA
REM OSA (broad) (*n* = 3,427)	2,391 (69.8%)	1,259 (36.7%)
REM-predominant OSA (strict) (*n* = 2,569)	1,838 (71.5%)	1,151 (44.8%)

Data are presented as n (%). Percentages are calculated within each row. POSA includes both supine-isolated and supine-predominant OSA. REM-predominant OSA analyses were restricted to patients with ≥ 30 min of REM sleep (*n* = 11,073)

Abbreviations: e-POSA, exclusive positional obstructive sleep apnea; p-POSA, predominant positional obstructive sleep apnea; REM, rapid eye movement

## Discussion

To the best of our knowledge, this is the study with the largest analyzed sample aimed to determine the prevalence of POSA among patients with OSA in Brazil, a country with one of the most highly miscegenated populations in the world. According to our data, 66.3% (two-thirds) of all patients with OSA presented POSA, a value consistent with previous studies (20–75%) [8, 13]. The prevalence of p-POSA and e-POSA reported in the literature has been observed at 50–67% and 25–32% of patients, respectively [14]. In our study, the prevalence of e-POSA was 27.8%.

The variability in prevalence found in the literature can be attributed to sample size, patient ethnicity and the classification system used for POSA, such as Cartwright’s system and the Amsterdam Positional OSA Classification (APOC) [15]. Regarding ethnicity, the Asian population has a higher prevalence of POSA compared to Western countries [16]. In terms of classification, Cartwright (1984) pioneered associating sleep position with OSA severity, classifying POSA when the AHI in the supine position was at least twice as high as in the non-supine position, finding a 50% prevalence of POSA in a sample of 24 patients [5]. The APOC was developed to identify patients who could benefit from positional therapy for POSA, adding to Cartwright’s criteria the requirement that the patient spends at least 10% of their sleep time in both the best and worst positions. Patients classified as POSA were divided into three groups, which guide treatment with exclusive or combined positional therapy. Patients were classified as APOC I if they had an AHI < 5 in the BSP; as APOC II if their AHI in the BSP corresponded to a lower OSA severity category compared with the overall AHI; and APOC III if they had an overall AHI ≥ 40 with at least a 25% reduction in AHI in the BSP. Patients with true POSA, categorized as APOC I, may be effectively treated with positional therapy alone. In contrast, patients classified as APOC II or APOC III may require a combination of therapeutic approaches [15].

As observed in OSA in general, POSA is more prevalent in men, with the degree of male predominance varying according to disease severity [17] However, as in other study, we found a higher prevalence of e-POSA in women (47.8% vs. 38.3%) [13]. This sex difference may be related to hormonal effects. Women tend to have a gynoid fat distribution pattern, with relatively less fat deposition in the upper airway, which may contribute to lower baseline collapsibility and a greater influence of positional factors on OSA severity [16].

Patients with p-POSA and e-POSA show significantly lower AHI and milder OSA compared to the non-POSA group. These patients also spent less time in N1 and N2 sleep and more time in N3 and REM sleep, which could lead to lower apneic arousal and total arousal indices compared to non-POSA patients [14]. In our sample analysis, it was observed that patients with POSA had lower AHI during both REM and NREM sleep, less time with oxygen saturation below 90%, higher mean oxygen saturation, reduced sleep fragmentation, higher amounts of N3 and REM sleep stages, and greater sleep efficiency. Importantly, several differences between patients with POSA and non-POSA persisted after adjustment for total AHI, particularly in measures of sleep architecture, fragmentation, and oxygenation, indicating that these findings are not solely explained by overall OSA severity. However, the magnitude of these differences was small, suggesting limited clinical impact. In contrast, some variables, such as NREM oxygen saturation and total arousals, were no longer significantly different after adjustment, suggesting that these parameters may be more directly driven by overall disease burden. In addition, significant group-by-AHI interactions indicated that these differences were more pronounced at lower AHI levels and attenuated with increasing severity. Together, these findings suggest that differences between POSA and non-POSA cannot be fully explained by severity alone, although their clinical relevance appears modest. One possible explanation is that POSA patients may experience periods of relatively preserved breathing when sleeping in non-supine positions, allowing partial recovery of sleep architecture and more stable oxygenation. In contrast, non-POSA patients may be exposed to a more continuous burden of respiratory events across sleep positions, leading to more sustained sleep disruption. Furthermore, our analysis revealed a higher prevalence of e-POSA in patients with mild POSA, while conversely, p-POSA was more prevalent in those with moderate and severe POSA.

In our large clinical sample, the prevalence of positional OSA was 66.3%. Pathophysiological mechanisms likely contribute to the high prevalence of positional OSA observed in clinical cohorts. Studies have shown that Pcrit decreases by about 1.8–2.5 cmH₂O in the lateral compared to the supine position, indicating reduced airway collapsibility [4, 17–20]. Camacho demonstrated in a case series that, in the supine position, the upper airway of OSA patients is significantly smaller than in the upright position, with a 32.6% reduction in total volume and an average 75.9% decrease in the minimal cross-sectional area [21]. Drug-induced sleep endoscopy findings indicate that body position significantly alters the pattern of upper airway collapse, with the supine posture favoring anteroposterior collapse of the velum, tongue, and epiglottis, consistent with gravity-dependent mechanisms, particularly in non-obese individuals [22].

The transition from upright to supine posture leads to a reduction in functional residual capacity, primarily driven by a decrease in expiratory reserve volume, which reduces caudal traction on the upper airway and increases pharyngeal collapsibility [16]. POSA patients, when compared to controls and REM-OSA patients, experience a greater reduction in awake lung functional residual capacity (in approximately 340 mL) when moving from the lateral to the supine position [23].

The impact of lung volume–dependent mechanisms may be particularly relevant in non-obese individuals, as expiratory reserve volume and functional residual capacity decline more markedly within the BMI range of 25–30 kg/m², which is typical of patients with positional OSA [24]. Supporting this concept, experimental animal study investigated postural responses in end-expiratory lung volume in mice and noted that only lean mice (but not obese mice) experienced a significant reduction in this lung volume when placed from prone to the supine position. The study assessed both end-expiratory lung volume and upper airway caliber, demonstrating that reductions in end-expiratory lung volume were associated with narrower upper airways, particularly in obese mice [25]. Consistent with these physiological observations, POSA patients in our cohort had significantly lower age and BMI compared to non-POSA, in line with previous studies [8]. The lower BMI observed in POSA patients may reflect a phenotype with greater positional dependence, which appears to decrease with increasing body weight. Supporting this dynamic model, weight loss appears to preferentially reduce nonsupine respiratory events compared to supine events, resulting in a relative increase in positional dependence and a shift from non-positional to POSA [26]. Conversely, longitudinal data from a Finnish cohort of 81 patients with POSA followed over 6 years indicate that weight gain was an important factor in developing NPOSA, with worsening AHI and oxygen desaturation [27].

The prevalence of REM-OSA has varied substantially among cross-sectional studies (17–74%), likely due to differences in diagnostic definitions and gender distribution [28]. Previous studies have demonstrated higher rates of events during REM sleep in the supine position; however, the overlap between POSA and REM-OSA has not been consistently characterized [29]. In our study, we observed a partial overlap between POSA and REM-OSA (1/3), whereas the inverse analysis showed that a substantial proportion of patients with REM-OSA also had POSA (approximately two-thirds). Although we did not directly quantify the events that occur during REM sleep in the supine position, as our classifications were based on global definitions related to REM and position, the observed overlap patterns suggest a possible interaction between sleep stage-dependent vulnerability and positional effects. However, this interpretation should be considered hypothesis-generating rather than confirmatory.

A limitation of some physiological studies is that key measures, such as lung volume and airway shape, are obtained during wakefulness, which may not fully capture the sleep state–dependent changes that contribute to airway collapse [23]. However, as previously discussed, the transition from an upright to a supine posture leads to a reduction in lung volume, decreasing caudal traction on the upper airway and increasing pharyngeal collapsibility [23]. In parallel, REM sleep, characterized by reduced muscle tone and alterations in ventilatory control, further increases vulnerability to upper airway collapse [28]. Although functional residual capacity appears to be similarly reduced in NREM and REM sleep in healthy individuals, evidence from patients at risk for OSA, particularly those with more severe disease, suggests a reduction in end-expiratory lung volume (EELV) during sleep, especially during REM sleep [30, 31]. These reductions in EELV may amplify the effects of the supine position on airway collapsibility in susceptible individuals. In this context, data from microgravity studies demonstrate a greater reduction in AHI during NREM sleep (68%) compared with REM sleep (30%), although both reductions are significant relative to normal gravitational conditions [32]. This finding suggests that REM-related events, while responsive to reduced gravitational effects, are also influenced by intrinsic neuromuscular vulnerability.

Thus, the high prevalence of POSA among patients with REM-OSA in our cohort suggests that these individuals may exhibit a combined phenotype, in which gravitational factors amplify an underlying REM-dependent upper airway instability. However, the partial overlap of REM-OSA among patients with POSA suggests that positional dependence cannot be fully explained by REM-related mechanisms alone. This asymmetric overlap between REM-OSA and POSA observed in our data highlights the importance of considering multiple interacting physiological traits in the characterization of OSA phenotypes.

This study has several strengths, including data analysis using Type 1 PSG (the gold standard test to diagnose OSA), and being, to our knowledge, the largest Brazilian sample studied to date for evaluating POSA prevalence. The analyses were stratified by sex and OSA severity categories, highlighting novel and relevant scientific findings. Some limitations should be acknowledged. We did not include clinical data such as neck circumference, associated comorbidities, and daytime symptoms. We were unable to use the thermistor sensor in some patients due to the COVID-19 pandemic, but still, the sample is representative. Additionally, the diagnosis of POSA was based on a single PSG exam, and it should be noted that POSA can be an unstable phenotype with night-to-night variability. The absence of directly measured sleep time in each body position represents another limitation of this study. Short durations of sleep in a given position may lead to unstable or unrepresentative positional AHI estimates, potentially resulting in misclassification of positional OSA, especially in the presence of REM-related effects. However, evidence regarding the representativeness of sleep position during polysomnography is mixed. While some data indicate that in-laboratory conditions may increase time spent in the supine position and potentially overestimate OSA severity, more recent data have shown similar supine sleep time between laboratory and home recordings, including analyses across hourly intervals throughout the night, suggesting comparable positional patterns at the group level [33, 34]. Therefore, our results are likely to reflect a typical night for patients at home. Importantly, in an exploratory sensitivity analysis using conservative plausibility criteria for positional exposure, prevalence estimates remained largely unchanged, supporting the robustness of the main findings despite the absence of directly measured positional sleep time. Although all studies were scored by experienced sleep specialists using standardized AASM criteria, formal inter-scorer reliability testing was not performed. Therefore, some degree of inter-scorer variability cannot be excluded.

## Conclusion

In a large clinical cohort, POSA was a highly prevalent phenotype, more common in younger, less obese individuals with milder OSA, and more frequent in women when exclusively positional. It showed a more favorable sleep and oxygenation profile independent of AHI. Differences in POSA, although of small magnitude, were more evident in milder disease and diminished with increasing severity. The overlap with REM OSA was asymmetric, with positional dependence being more frequent in REM OSA than the converse, supporting the concept of distinct but partially overlapping phenotypes. Overall, POSA represents a heterogeneous phenotype, underscoring the need for individualized approaches to OSA management.

## Data Availability

All data files are available.
